# Supplementary material for: Cost-Effectiveness Analysis of Personalized Hypertension Prevention
Source: J Pers Med. 2023 Jun 15;13(6):1001. doi: 10.3390/jpm13061001 (PMC10302947; doi:10.3390/jpm13061001)
Supplement: Supplementary file 1 [file jpm-13-01001-s001.zip › jpm-2424221-supplementary.pdf]

Supplementary Table S1. Prevalence of prehypertension (120<=SBP<140 mmHg or 80<=DBP<90 mmHg or history of hypertension) by age and gender.

Age group	Male		Female		Total	
	Cases with total samples in parentheses	Prehypertension Prevalence (%)	Cases with total samples in parentheses	Prehypertension Prevalence (%)	Cases with total samples in parentheses	Prehypertension Prevalence (%)
20–29	3133(6285)	49.8	1893(10060)	18.8	5026(16345)	30.7
30–39	5104(9896)	51.6	4328(17345)	25.0	9432(27241)	34.6
40–49	5826(11362)	51.3	6540(18320)	35.7	12366(29682)	41.7
50–59	4283(9096)	47.1	5770(14141)	40.8	10053(23237)	43.3
60–69	3121(7505)	41.6	3839(9462)	40.6	6960(16967)	41.0
70–79	2036(5408)	37.6	1701(4649)	36.6	3737(10057)	37.2
Total	23503(49552)	47.4	24071(73977)	32.5	47574(123529)	38.5

Supplementary Table S2. Prevalence of stage1 hypertension (141<=SBP<160 mmHg or 90<=DBP<100 mmHg or history of hypertension) by age and gender.

Age group	Male		Female		Total	
	Cases with total samples in parentheses	Stage1 Hypertension Prevalence (%)	Cases with total samples in parentheses	Stage1 Hypertension Prevalence (%)	Cases with total samples in parentheses	Stage1 Hypertension Prevalence (%)
20–29	353(6287)	5.6	136(10061)	1.4	489(16348)	3.0
30–39	970(9897)	9.8	540(17354)	3.1	1510(27251)	5.5
40–49	1810(11367)	15.9	1680(18327)	9.2	3490(29694)	11.8
50–59	2488(9099)	27.3	3090(14149)	21.8	5578(23248)	24.0
60–69	2797(7514)	37.2	3283(9479)	34.6	6080(16993)	35.8
70–79	2410(5426)	44.4	1995(4673)	42.7	4405(10099)	43.6
Total	10828(49590)	21.8	10724(74043)	14.5	21552(123633)	17.4

Supplementary Table S3. Prevalence of stage2 hypertension (160<=SBP or 100<=DBP or history of hypertension) by age and gender.

Age group	Male		Female		Total	
	Cases with total samples in parentheses	Stage2 Hypertension Prevalence (%)	Cases with total samples in parentheses	Stage2 Hypertension Prevalence (%)	Cases with total samples in parentheses	Stage2 Hypertension Prevalence (%)
20–29	26(6287)	0.4	11(10061)	0.1	37(16348)	0.2
30–39	95(9897)	1.0	72(17354)	0.4	167(27251)	0.6
40–49	240(11367)	2.1	195(18327)	1.1	435(29694)	1.5
50–59	313(9099)	3.4	300(14149)	2.1	613(23248)	2.6
60–69	387(7514)	5.2	325(9479)	3.4	712(16993)	4.2
70–79	299(5426)	5.5	253(4673)	5.4	552(10099)	5.5
Total	1360(49590)	2.7	1156(74043)	1.6	2516(123633)	2.0
